# The evolutionary trajectories of specialized metabolites towards antiviral defense system in plants

**DOI:** 10.1186/s43897-023-00078-9

**Published:** 2024-01-12

**Authors:** Naveed Ahmad, Yi Xu, Faheng Zang, Dapeng Li, Zhenhua Liu

**Affiliations:** 1 Joint Center for Single Cell Biology, Shanghai Collaborative Innovation Center of Agri-Seeds, School of Agriculture and Biology, Shanghai Jiao Tong University, Shanghai, 200240 China; 2 Department of Plant Pathology, Nanjing Agricultural University, Nanjing, 210095 China; 3 Key Laboratory of Soybean Disease and Pest Control (Ministry of Agriculture and Rural Affairs), Nanjing Agricultural University, Nanjing, 210095 China; 4 National Key Laboratory of Advanced Micro and Nano Manufacture Technology, Shanghai Jiao Tong University, Shanghai, 200240 China; 5 National Key Laboratory of Plant Molecular Genetics, CAS-JIC Centre of Excellence for Plant and Microbial Science, Center for Excellence in Molecular Plant Sciences (CEPMS), Chinese Academy of Sciences, Shanghai, 200032 China

**Keywords:** Specialized metabolism, Plant-virus-insect interaction, Chemodiversity, Co-evolution

## Abstract

Viral infections in plants pose major challenges to agriculture and global food security in the twenty-first century. Plants have evolved a diverse range of specialized metabolites (PSMs) for defenses against pathogens. Although, PSMs-mediated plant-microorganism interactions have been widely discovered, these are mainly confined to plant-bacteria or plant-fungal interactions. PSM-mediated plant-virus interaction, however, is more complicated often due to the additional involvement of virus spreading vectors. Here, we review the major classes of PSMs and their emerging roles involved in antiviral resistances. In addition, evolutionary scenarios for PSM-mediated interactions between plant, virus and virus-transmitting vectors are presented. These advancements in comprehending the biochemical language of PSMs during plant-virus interactions not only lay the foundation for understanding potential co-evolution across life kingdoms, but also open a gateway to the fundamental principles of biological control strategies and beyond.

**Graphical Abstract:**

The putative co-evolutionary triad of plant metabolites (PSM) mediated interactions between plant, viruses and their insect vectors.

This dynamic trio is depicted through the interplay represented by pink and green arrows, signifying the PSM mediated bidirectional interactions occurring between the virus, the host plant, and the vector.

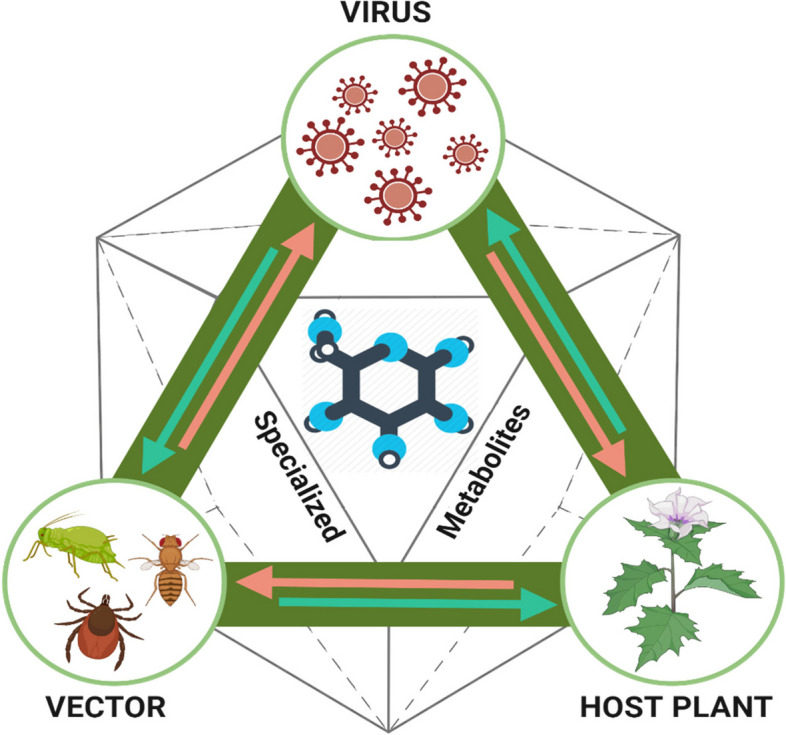

## Introduction

Plant viruses are obligate and biotrophic parasites that are dependent on host cellular machinery for their replication and transmission. The genomes of these viruses are typically small (ranging from 2 to 20 kilobases), containing DNA and/or RNA that encode a variety of essential proteins, such as the coat protein, movement protein, and replication-associated enzymes, along with a range of less-conserved proteins (Koonin et al. 2021). Alas, their detrimental effects lead to serious plant diseases, causing significant losses in yields across important crop species. This poses a global threat to food security and agricultural productivity, especially for the ever-increasing world population (Awasthi et al. 2016). Approximately, 15% of global crop production is devastated by plant diseases, and viruses alone account for one-third of these losses (Boualem et al. 2016; Yadav and Chhibbar 2018). Most viruses elicit severe symptoms leading to multiple physiological disorders in plants, threatening agriculture productivity and yield (Lefeuvre et al. 2019). However, some viruses cause symptomless or mild disease with no appreciable effect on plant growth and/or yield (Takahashi et al. 2019). The development of next-generation sequencing (NGS) technologies has significantly enabled the identification and characterization of plant viruses. Currently, more than 2,100 plant virus species have been approved by the International Committee on Taxonomy of Viruses (Walker et al. 2021).

**Fig. 1:**
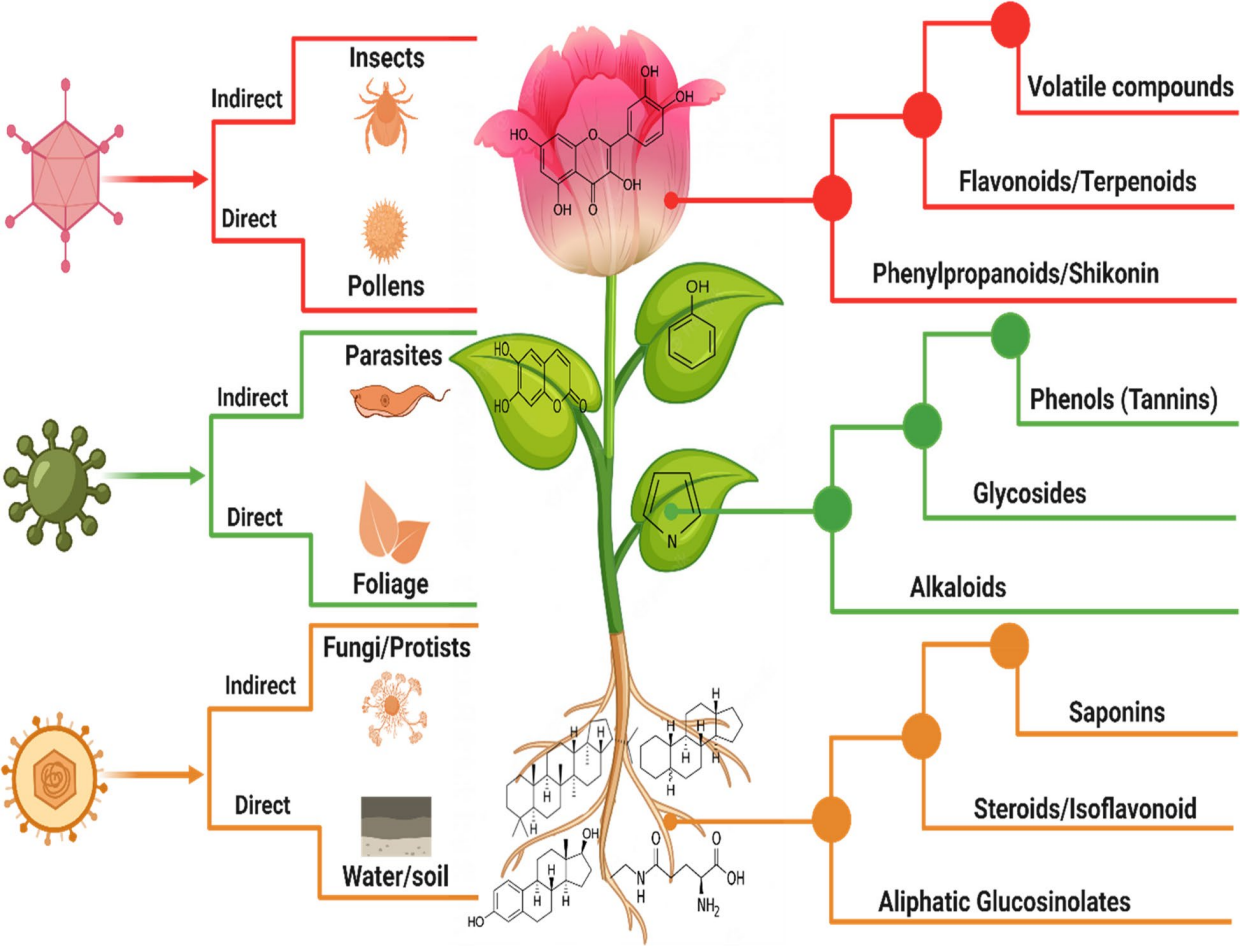
Viral transmission pathways and major classes of PSMs across different tissues

Plant viruses naturally developed an arsenal of transmission pathways, providing the medium for their plant-to-plant spread (Fig. 1). On one hand, they use direct (mechanical) methods, such as physical contact through agricultural activities, contaminated soil, water, or human touch (Jones 2018). Even the intimate process of pollination can unwittingly become a viral transmission route in flowering plants (Hull 2013). On the other hand, their reach extends through indirect pathways (horizontal) involving vectors like aphids, wind-blown mites, whiteflies, and nematodes (Jones and Naidu 2019). These microscopic worms carry viruses within their tubular mouthparts and inject them into plant vascular tissues. In response, plants have evolved sophisticated mechanisms of antiviral defense system such as RNA interference, hormonal signaling and leveraging their metabolic prowess to synthesize specialized metabolites (Calil and Fontes 2017). However, within this complexity, the specialized synthesis of biochemical substances aimed at countering viral pathogens often remains poorly explored. A diverse range of chemical compounds are produced by plants, commonly known as PSMs (Weng et al. 2012). The exact number of these PSMs across the plant kingdom remains unknown (approximately over 200,000) as more plant species are to be examined at the molecular level. These metabolites exhibit an incredible chemical diversity, comprising phenolic compounds, alkaloids, terpenoids and many more. In addition to their vast chemical diversity, PSMs also accumulate in diverse plant organs, showcasing qualitative and quantitative variations across plant species and different organ types. Additionally, the accumulation profiles of PSMs vary across developmental stages, with significant variations between seedlings and those in mature flowering or aging plants (Wink 2010).

Metabolites-mediated plant-microorganism interactions have been widely discovered (Huang et al. 2019; Zhong et al. 2022), however, these are mainly confined to plant-bacteria or plant-fungal interactions, leaving antiviral defense system in plants largely untapped. As obligate intracellular pathogens, the replication and spread of viruses depend exclusively on vectors and the host cell machinery, posing complex challenges in discoveries of chemical-mediated plant-virus interactions. A plausible arms race between plants and viruses has likely facilitated fast evolution of both virus and PSMs, adding additional barriers in understanding the chemical defense against viruses. However, the PSM-mediated defense and counter-defense mechanisms of plants and viruses continues to emerge recently, reflecting their putative co-evolutionary relationship (Wu et al. 2019; Gong et al. 2023). As suggested by previous studies, viral infection effeciently induced metabolic changes and increased activities of antioxidant enzymes in several crop species (Vega et al. 2011; Lan et al. 2020). It was also reported that the induction of hypersensitive response (HR) in the Arabidopsis turnip crinkle virus (TCV)-resistant line, which is regulated by a singular dominant nuclear locus known as HRT tightly linked to dihydroflavonol 4-reductase (DFR) locus. This study further showed an increased accumulation of salicylic acid, camalexin, and autofluorescent cell-wall material in the TCV-resistant line in comparison to susceptible lines (Dempsey et al. 1997). These studies suggest the significance of PSMs in proposing emerging strategies of defence and counterdefence mechanisms between plants and viruses in the future.

In this review, we aim to scratch the surface of the plant metabolic space, with a particular focus on the evolution of specialized metabolites and their roles against plant viruses. We also comprehend the astounding diversity of the PSMs and their implications on plant-virus-vectors dialogue, and highlight the profound depths of their ongoing evolutionary arms race. This captivating molecular saga not only underscores the complexity of plant-virus interactions but also offers a treasure trove of insights for developing new metabolic engineering strategies against viral foes and reinforcement of crop protection and global food security.

### Plant chemodiversity and their antiviral activities

**Fig. 2:**
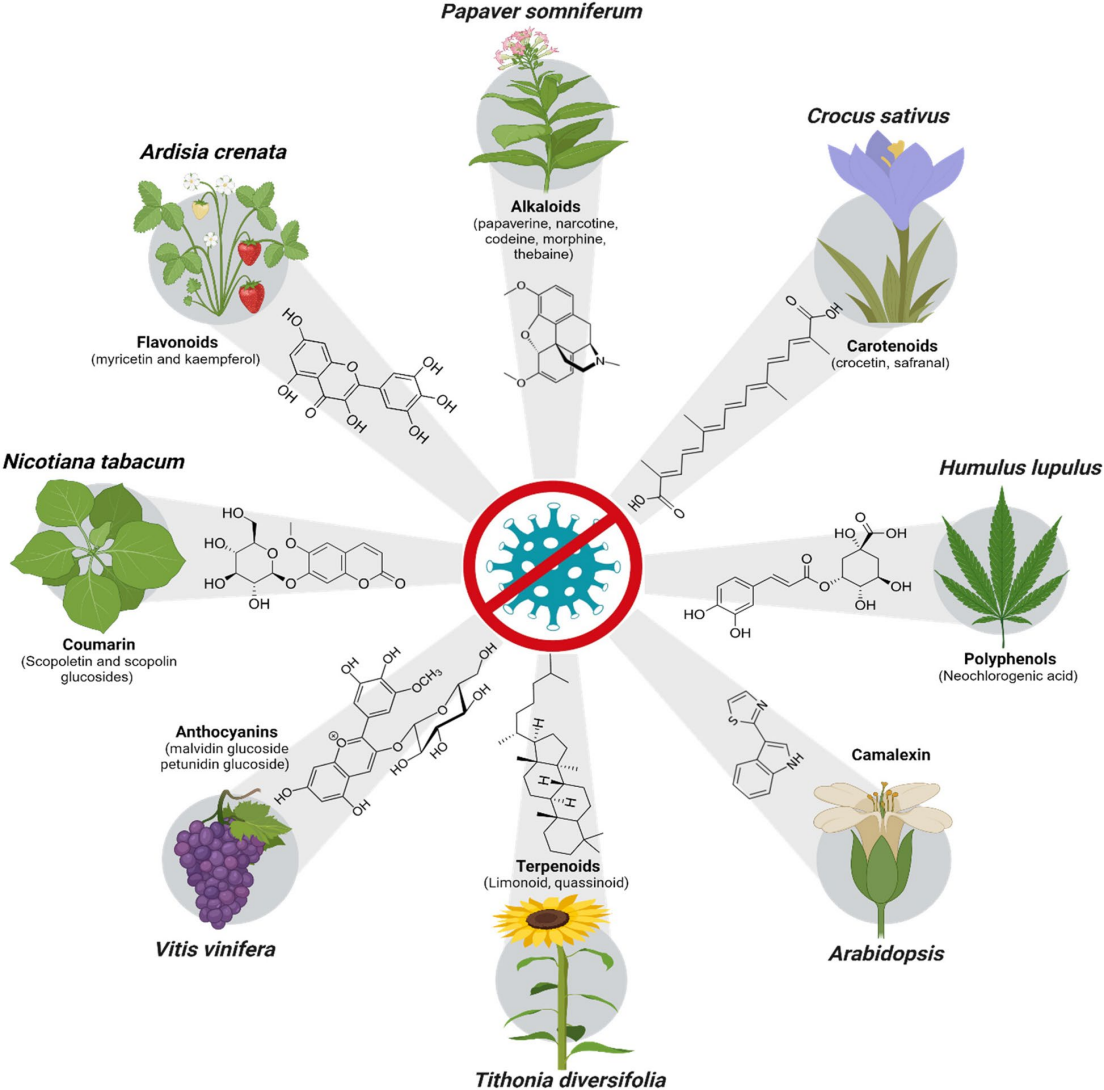
Antiviral specialized metabolites of plant origin. The diverse classes of specialized metabolites alongside their chemical structures identified with antiviral resistance against different plant viruses (TMV, CMV, PMV-P, PVX and PVY) were illustrated with their respective plant species. These metabolites exemplify the fascinating array of plant defenses, shedding light on their potential as sources of antiviral agents during plant-virus interactions

Plants speak a chemical-encoded language, enabling dialogues between plant and other organisms. For instance, many plants release volatile organic compounds (VOCs) to attract pollinators or repel herbivores. Many PSMs are demonstrated as toxins, attractants or mutualistic signals in the model plants such as *Arabidopsis* and tobacco (Kessler and Baldwin 2002). Some PSMs exhibit direct virucidal activity by inhibiting viral attachment or inactivating viral particles. Others interfere with viral replication by targeting viral enzymes or disrupting essential viral proteins and inhibiting key steps in their replication process (Zhou et al. 2018; Dixit et al. 2020; Lan et al. 2020). In the following section, we presented a wide range of such examples across different classes of PSMs identified with antiviral properties (Fig. 2).

#### Phenolic compounds

Phenolic compounds are a large and diverse group of organic compounds that contain a phenolic ring structure, mainly derived from the two aromatic amino acids (phenylalanine and tyrosine) in plants. Through a series of biochemical conversions (e.g. deamination, hydroxylation, methylation and glycosylation), they are diversified into > 8,000 structurally and functionally diverse small molecules (Patil and Masand 2018). Phenylpropanoids, a class of phenolic compounds, constitute the major component of lignin as supporters of cell walls, which serve as "physical barriers" to defend plants against bacterial and fungal pathogens. However, whether the thickness of plant cell walls shield against plant virus remains an open question. Flavonoids, an important branch of phenylpropanoids, are the main contributors of color, flavor, and aroma in plants (Ahmad et al. 2020, 2023; Hong et al. 2023). Different types of flavonols such as (myricetin, kaempferol, and quercetin derivatives) as well as hydroxycinnamic acids (including caffeic acid derivatives) were induced by GLRaV-3 infection in the leaves of white berry cultivar (*Malvasía de Banyalbufar*) (Montero et al. 2016). Similarly, the red-skinned berries (*Vitis vinifera* cv. Zinfandel) following infection with *Grapevine red blotch-associated virus* (GRBaV) during berries ripening also suggested modulation in their secondary metabolic flux including flavonoid and anthocyanin biosynthesis (Blanco-Ulate et al. 2017). Furthermore, tobacco (Nicotiana spp.) and tomato (*Solanum lycopersicum* L.) crops during TMV infection also showed abundance in phenolic compounds including 5-O-caffeoylquinic acid and quercetin at the TMV infection sites (Choi et al. 2006). These findings provide important insights into the induced accumulation of both central and peripheral phenylpropanoids upon viral infection, indicating co-association and co-interaction of both virus and specialized metabolism, which can be utilized for potential tailored strategies to overcome viral infections.

At the molecular level, maize phenylalanine ammonia-lyases (ZmPALs, the first committed step of the phenylpropanoid pathway) have been shown to play a crucial role in conferring resistance against SCMV infection by positively regulating salicylic acid (SA) accumulation and Pathogenicity-related (PR) gene expression (Yuan et al. 2019). In addition, ZmPALs were also shown to be involved in SCMV-induced lignin accumulation, highlighting their contribution in limiting virus accumulation and moderating symptom severity. In soybean, CRISPR/Cas9-mediated gene mutagenesis of flavanone-3-hydroxylase (F3H) and flavone synthase II (FNS II) led to increased isoflavone content and improved resistance to soybean mosaic virus (Zhang et al. 2020). Other types of phenolic compounds such as Gramniphenol C, Gramniphenol F, and Gramniphenol G isolated from *Arundina gramnifolia* have been identified with significant antiviral activity against TMV (Hu et al. 2013). Overall, the accumulation of phenylpropanoids in plants has likely provided a formidable antiviral defense system, offering a diverse array of mechanisms to combat viral infections and mitigate their impact on plant health. Nevertheless, the specific effectors and physiological processes that contribute to these unforeseen consequences are still being investigated and have not been comprehensively elucidated.

#### Alkaloids

Alkaloids are a diverse class of naturally occurring phytochemicals, which share the common feature of containing at least one nitrogen atom, usually in a heterocyclic ring. They comprise a repertoire of roughly 20,000 known compounds, primarily synthesized from amino acid precursors like tyrosine, lysine, ornithine, phenylalanine, and tryptophan (Desmet et al. 2021). These precursors are transformed into a variety of core intermediates, which lead to production of various chemical forms, exhibiting structural and functional diversity. Previous studies have explored that the infection of opium poppy (*Papaver somniferum*) plants with PMV-P virus has significantly induced the accumulation of different types of alkaloids. Notably, PMV-P infection exhibited a genotype-dependent impact on the accumulation level of different alkaloids, leading to altered profile of papaverine, thebaine, narcotine, codeine, and morphine (Zaim et al. 2011, 2014). These investigations propose a significant role of alkaloids in reducing PMV-P infection, suggesting their possible use in modulating plant defense responses. However, the specific mechanisms of action vary depending on the type of alkaloid and the targeted viral species. Prior studies also suggested that indole, a core backbone structure of many alkaloids has been widely used in the discovery of antiviral activities. For instance, the indole-derived compound 2,4-bis (5-bromo-1H-indol-3-yl) thiazole has been shown to impede the movement of TMV by exhibiting certain antiviral properties such inactivation, curative, and protective effects (Guo et al. 2019). Similarly, nortopsentin alkaloids have been demonstrated to confer anti-TMV activity by inhibiting the assembly of viral particles (Ji et al. 2018). Similarly, the antiviral potential of the indole and thiol derived compounds also suggested remarkable efficacy against PVY, CMV, and TMV-typed viruses. It was further confirmed that the antiviral activity of these compounds was closely linked to a surge in chlorophyll content and the increased activity of defense-related enzymes (Wei et al. 2019).

The molecular mechanism of nitrogen-containing heterocyclic compounds containing indole skeletons such as topsentin, harmine and gramine derivatives suggested their antiviral potency targeting TMV (Lu et al. 2019). It has been shown that these compounds impede the assembly of TMV by cross-linking the viral capsid protein, reshaping viral structures, promoting the activity of antioxidant enzymes and limiting the production of TMV-induced ROS during plant infection. It also triggers an upsurge in salicylic acid levels by regulating the expression level of *PR2* response gene (Lv et al. 2021). Moreover, their impact on tobacco growth and biomass accumulation was contingent on concentration, showcasing a nuanced relationship (Zhang et al. 2021). In addition, the compound 7-deoxytrans-dihydronarciclasine, an alkaloid isolated from the *Hosta plantaginea* has also indicated resistance against TMV (Wang et al. 2007). Similarly, Bruceine-D, identified within the extract of *Brassica javanica* exhibits inhibitory effects on PVY, CMV, and TMV infections (Shen et al. 2015). Altogether, these findings not only revealed important insights into the pivotal role of alkaloids towards the discovery of novel antiviral leads but also warrant future researches on their evolutionary crossroads during plant-virus interaction.

#### Terpenoids

Terpenoids, also known as isoprenoids, are the most widespread and structurally diverse class of natural products found in many plant species. Further classifications of terpenes are based on the quantity of isoprene units (C5) present in their structure, such as monoterpene (C10), sesquiterpene (C15), diterpene (C20), triterpene (C30), and so forth. They are mainly derived from the five-carbon building blocks of isopentenyl diphosphate (IPP) and dimethylallyl diphosphate (DMAPP) through either the mevalonic acid (MVA) in the cytosol or the 2-methyl-D-erythritol 4-phosphate (MEP) pathway in plastids (Tholl and Lee 2011). Terpenoids are mainly associated with important roles in regulation of growth and development, pigments, hormonal signaling, photosynthesis and transport. Furthermore, due to the volatile property of low-molecular weight terpenoids and terpene-derived phytoalexins, they are known to attract/repel pollinators and/or herbivores, thereby mediating antagonistic and beneficial interactions, defending plants against predators, pathogens and competitors (McCormick et al. 2012). Hence, these chemical compounds are being considered crucial in resistance to diseases caused by bacteria, fungi and even viruses. For example, there is a notable inverse relationship between the amount of the diterpenoid phytoalexin momilactone A that accumulates in rice and the degree of white-backed planthopper (*Sogatella furcifera*) infestation, which is a well-known transmission vector of SRBSDV (Zhou et al. 2013). This referenced correlation highlights the potential of diterpenoid phytoalexins as promising chemical compounds exhibiting anti-herbivore effects (Kanno et al. 2012). Likewise, parallel findings also indicated the efficient role of triterpenoids (quassinoids) as effective antiviral agents against TMV infection (Yan et al. 2010). Previously, it was found that the *Picrama quassioides* wood extract exhibited strong anti-TMV effects. During this investigation, promising compounds of quassinoids and β-carboline alkaloids were discovered, revealing that when β-carboline was combined with quassinoids, it resulted in a higher rate of inhibiting TMV infection compared to β-carboline alone (Chen et al. 2009). The antiviral activities of certain oxygenated limonoids have been shown to inhibit the TMV infection in *Munronia unifoliolata* olive plant (Ge et al. 2012).

Enzymatic activity and genetic evidence of a sesquiterpenoid known as capsidiol and/or capsidiol 3-acetate also shown antiviral responses, providing resistance against TMV and PVX in tobacco plants (Li et al. 2015). Researchers also found that a labdane-type diterpene called WAF-1 functions as an internal signal that switches on the defense response against TMV in tobacco plants (Seo et al. 2003). Similarly, a recent finding discovered four novel triterpenoids, known as Toosendansins (A-D), from the fruits of *Melia toosendan*, indicating anti-TMV activities along with the ability to protect SH-SY5Y cells against H_2_O_2_-induced damage during infection (Chen et al. 2014). New natural roles undoubtedly remain to be discovered for this large class of compounds, given that such a small percentage of terpenes has been investigated so far. As researchers continue to investigate the intricate biochemical pathways and interactions within various plant species, it is highly plausible that more instances of triterpenoids with remarkable antiviral activities will emerge, further expanding the domain of potential antiviral applications.

#### Other natural compounds

The discovery of new antiviral chemical compounds (other than flavonoids, alkaloids, and terpenoids) further highlights the significant role of natural products in the search for effective antiviral remedies. For example, natural fatty acids and polysaccharides have shown notable efficacy against certain plant viruses (Wang et al. 2013; Deshoux et al. 2020). Recently, it has been shown that fructo-oligosaccharide from greater burdock (*Arctium lappa*) exhibited anti-TMV activity and enhanced the expression of *PAL*, *PR-1* and *PR-5* genes in tobacco plants. The anti-plant virus potential of this natural compound seems intricately linked with the expression and activation of diverse defense-associated genes in tobacco (Zhao et al. 2017). Several coumarin derivatives, including those with dithioacetal and sulfonamide structures, have shown promise in combatting CMV infection by influencing enzyme activities related to plant defense mechanisms and chlorophyll levels. They achieve this modulation by interacting with the abscisic acid (ABA) pathway in tobacco plants (Zhao et al. 2022). In light of these discoveries, the investigation and identification of other natural compounds could further inspire high-throughput metabolite engineering strategies for tailored antiviral strategies in the future.

### Evolutionary scenarios for metabolites-mediated interactions between plant, virus and virus-transmitting vectors

Plant virus largely depend on vectors for transmissions, approximately 80% of them rely on insects (Hohn 2007). In response, plants synthesize ubiquitous specialized metabolites that serve as signaling molecules, providing recognition cues for the identification and colonization of these organisms, particularly insects (Pickett and Khan 2016). Understanding about how these three-way relationships have been shaped and evolved will lead to the effective management of current diseases and proactive prevention of future pandemics. In this section, we will focus on metabolites mediated interactions between plant, virus and virus-spreading vectors, particularly insects.

Plants exhibit an impressive capacity to produce a wide array of natural chemicals, each possessing its distinct structure and activity, primarily functioning as defensive compounds. However, the evolutionary processes that have led to this astounding metabolic diversity at the molecular level remain less understood. One possible mechanism that gene and genome duplications, followed by natural selection-driven sub- or neo-functionalization is widely accepted for enzymatic innovations in plants (Weng et al. 2012). Recently, metabolic gene clustering and horizontal gene transfer (HGT) were interpreted as new metabolic diversity drivers (Nützmann et al. 2016; Wu et al. 2022a, b; Zhou and Liu 2022). Furthermore, co-evolution with herbivores and pathogens can act as a driving force for the diversification of PSMs. For example, the coevolutionary relationship between butterflies and Brassica plants is exemplified by the development of sulfur-containing glucosinolates (GLS). This suggests that enhanced GLS complexity prompted butterflies to evolve countertactics, enabling them to persist in attacking and feeding, leading to an ongoing cycle of adaptation. Likewise, the occurrence of co-divergence between tobamoviruses and angiosperms plants were also identified (Gibbs et al. 2015). The study highlights the coevolutionary relationship between tobamoviruses and their eudicotyledonous hosts, demonstrating that most tobamoviruses, except those infecting brassicas, likely co-diverged with their hosts around 112.9 million years ago.

**Fig. 3:**
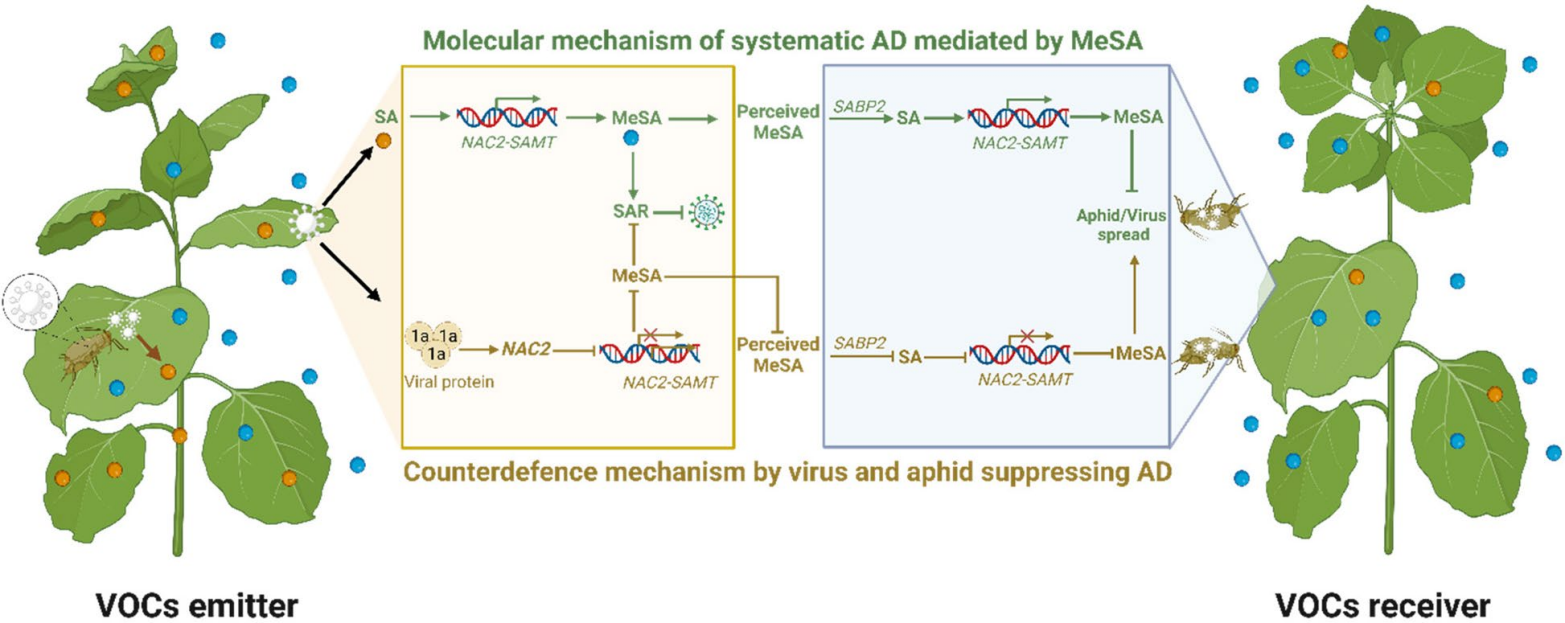
A recent reported example of PSM-mediated plant–insect-virus interactions. During the airborne defense mechanism, VOC-emitter plants sense aphid sap-sucking action, triggering the biosynthesis of SA. The *NAC2*-*SAMT1* module is then activated to produce volatile MeSA. Receiver plants in the vicinity perceive MeSA and convert it into SA through the action of SABP2. SA functions as a cue, initiating the *NAC2*-*SAMT1* module in the receiver plants, consequently eliciting a defense response against both aphids and viruses. At the counterdefence mechanism, the aphid-transmitted virus *CMV*, employ their helicase-containing viral protein (e.g., CMV1a) to subcellularly re-localize and destabilize *NAC2*. This manipulation suppresses *NAC2*-mediated airborne defense, facilitating aphid propagation and virus transmission. As a result, plants become less repellent to aphids, generating a more favorable environment for aphid survival, infestation, and subsequent viral transmission. The figure is created with biorender.com and the illustration is adapted from the recent work of (Gong et al. 2023)

Although PSMs have been extensively shown to have synergistic or antagonistic effects on herbivores, the identification of specific compounds is often challenging due to their low abundance and the complexity of plant metabolism. Recently, researchers employed natural history-guided omics techniques in a native habitat, involving a 26-parent recombinant inbred line population alongside native herbivores. Their research identified a novel caffeoyl putrescine-green leaf volatile (CPH) compound in wild tobacco plants, which imparts nonhost resistance to Empoasca leafhoppers–notorious vectors responsible for the transmission of numerous viruses (Bai et al. 2022; Mohan et al. 2022). This seminal work also demonstrated that biosynthesis of the chemical weapon (CPH) is the output of the plant well-conserved JA-signaling pathway, which has been a common target manipulated by both plant virus and their insect vectors (Wu and Ye 2020). Remarkably, some plants can also communicate with the neighboring plants by emitting volatile compounds (VOCs) when encountered by insect attack. For example, Methyl-salicylate (MeSA) is an aphid-inducible VOC produce by plants (Babikova et al. 2013; Saad et al. 2015; Moreira et al. 2018). Very recently, a group of researchers revealed that MeSA could be perceived and converted into SA by salicylic acid-binding protein-2 (SABP2) in neighboring plants. Salicylic acid then causes a signal transduction cascade (NAC2-SAMT1) to promote MeSA biosynthesis to induce plant anti-aphid immunity and reduce virus transmission (Gong et al. 2023). This work further revealed that some aphid-transmitted viruses have evolved helicase-containing proteins to subcellularly re-localize and physically destabilize the transcriptional factor *NAC2*, resulting in suppressing the plant biosynthesis of MeSA (Fig. 3). The above cases thus represent excellent examples illustrating the PSMs-mediated interactions between plants, insects and viruses.

Besides VOCs, non-volatile metabolites are constantly reported to mediate plant–insect interactions as well, which likely impact the tripartite communication of plant, virus and insects (Luo et al. 2023). While plants actively defend themselves with PSMs, on the other hand, insects and their associated virus strains have also evolved counteradaptations strategies to avoid, tolerate, and detoxify them, consequentially favoring the transmission of plant virus. Previously, it has been shown that a plant-derived phenolic glucoside *malonyltransferase* gene (*BtPMaT1*), which is horizontally transferred into whitefly for detoxifying plant toxins (PSMs) (Xia et al. 2021). This important work highlights the underestimated impacts of HGT in exchanging metabolic genes between plants and insects. Intriguingly, whiteflies (family Aleyrodidae; Hemiptera) can transmit many viruses to plants, and several cases of HGT were found in this species, including the first tentative example of plant-to-insect HGT (Chen et al. 2016; Gilbert and Maumus 2023). Importantly, viruses are considered as important vectors of horizontal transfer of genetic material in eukaryotes (Gilbert and Cordaux 2017; Gilbert and Maumus 2023). Therefore, the impacts of plant virus on plant genomes and their encoded metabolism are likely underestimated. Some studies have also demonstrated that the virus seems capable of manipulating insect behavior including their feeding habits, in a manner that promotes successful infections in plants (Ingwell et al. 2012; Gutiérrez et al. 2013; Safari et al. 2019; Lee et al. 2022). However, the extent of PSM involvement in this complicated process still remains unclear.

From evolutionary point of view, there are several features shared between plant virus and PSMs. They are both largely intertwined with insect species, which presumably have facilitated their diversity. It is well known that PSMs are rapidly evolved during the sessile adaptation, so do plant viruses. This is because plant viruses typically have high mutation rates, relatively short reproductive cycles, common genetic recombination and the ability to spread horizontally. Unlike PSMs, the fast evolution of plant viruses sometimes caused significant losses in plants. For instance, the outbreak of soybean stay-green syndrome with delayed leaf senescence (stay-green) has swept the soybean production in the Huang-Huai-Hai region of China, resulting in huge yield losses (Xu et al. 2019). This virus is newly evolved from recombination events between different genera of geminiviruses (Cheng et al. 2022). However, it is sparsely understood that why and how plant viruses have facilitated the chemical diversity in plants (with or without the help of insect vectors). Meanwhile, it is still a considerable speculation that PSMs drive the co-evolution of plants and virus (likely also involving the vectors). With advances in isolating, detecting and analyzing of trace amounts of plant PSMs and virus from a highly mixture population of species (including spreading vectors), we will be able to understand these seemingly ephemeral equilibrium triple relationships in the near future.

## Conclusions and future perspectives

The adaptation of plants to land was a major breakthrough toward meeting the requirement to synthesize and diversify specialized metabolites. These metabolites, with their versatile chemical structures and properties, likely have enabled their host plants to interact with other organisms, including plant virus and virus-spreading vectors, particularly insects. Various classes of PSMs including phenolics, terpenoids, alkaloids are found to possess antivirus activities. However, this is a general observation, and in real situation, it is likely that certain types or a single PSM may only confer resistance to specific counterpart(s) of the virus. Interaction between plant and plant virus is more complicated than other mutualistic interactions mainly because of the additional involvement of spreading vectors. Interpreting evolutionary trajectories of PSM-mediated plant-virus interaction thus, in most of the cases, need to consider a three-way co-evolutionary mutualism. Up to date, only a tiny number of metabolites-mediated plant-virus interactions are reported. This is likely the tip of iceberg compared to the large quantity of both PSMs and viruses. Several intrinsic and extrinsic factors may have affected the discovery of the chemical-mediated mutualism between plant and virus. Overcoming these challenges, especially when dealing with the often trace amounts of both PSMs and plant viruses, necessitates the development and implementation of more advanced molecular and analytical techniques.

To bridge the gap between these observations on plant-virus interactions and the challenges of synthesizing plant-derived metabolites, it is worth noting that the majority of PSM biosynthetic pathways remain elusive, despite recent advancements in elucidating complex terpenoid pathways (Pichersky and Raguso 2018; De La Peña et al. 2023; Reed et al. 2023). Using a plant-based system to produce valuable plant-derived metabolites, the transient expression system of *N. benthamiana* is usually favored due to its amenability of expressing plant genes and metabolic support (Reed and Osbourn 2018). To this date, *N. benthamiana* has successfully facilitated the reconstitution of diverse PSM biosynthetic pathways such as alkaloids (Miettinen et al. 2014), glucosinolates (Crocoll et al. 2016), cyanogens (Rajniak et al. 2015), betalains (Polturak et al. 2016) and more. Although, microbial heterologous expression system has long been considered useful due to their efficient genetic manipulability and rapid replication, the agroinfiltration system of *N. benthamiana* has emerged a popular toolbox for reconstituting PSM pathways in plants. This preference for the plant based transient expression system over conventional microbial platforms is driven by its compatibility with mRNA and protein processes, cellular compartmentalization, and the presence of essential metabolic precursors and cofactors (Stephenson et al. 2020). Similarly, the emergence of next-generation sequencing technologies has greatly advanced plant virus metagenomics approaches (Roossinck 2012) and natural history-guided multi-omics frameworks for the exploration of PSMs that possess antiherbivore and antiviruses function (Bai et al. 2022). In addition, rapid tests of plant virus presence using immunoassays are making it possible to perform such virus evaluations on-site (Culver et al. 2015; Kalimuthu et al. 2022). Increase numbers of analytical tools in conjunction of advanced assay formats and state-of-the-art optical and electrical micro-/nano-transducers are contributing to fast, quantitative, yet cost-effective plant virus early identification and precise quantification (Jablonski et al. 2021; Dutta et al. 2022). These powerful tools offer the promise of developing future crop protection strategies that could be extended in a deeper understanding of plant-virus interactions.

Unlocking the vast chemical diversity of PSMs and unraveling the complexity of their biosynthetic pathways presents a remarkable opportunity to enhance the defense mechanisms of crop plants through advanced metabolic engineering techniques. These endeavors offer a promising avenue for the discovery of novel antiviral compounds, capable of targeting both viruses and their associated vectors. With cutting-edge metabolic engineering platforms, several distinct strategies can be proposed including the transportation of novel or exogenous biosynthetic pathways from distant species, reconfiguring existing metabolic pathways by manipulating gene expression, incorporating biosynthetic genes from closely related species and wild relatives, and harnessing regulatory switches such as transcription factors and miRNAs. Such and more of the same strategies may geared new frontiers toward strengthening the resistance of crop plants against viruses and particularly insect-transmitted viruses. As scientific progress continues, our understanding of the multifaceted roles of PSMs as defense regulators, inhibiting pathogenic microbes, while also operating as signaling molecules to attract insects, facilitating host–pathogen communication, and orchestrating a range of biological phenomena. The effective use of these deterrent metabolites could potentially pave the way for improving sustainable agriculture production and ecosystems alike.

## Data Availability

Not applicable.

## Abbreviations

ABA: Abscisic acid
CMV: Cucumber mosaic virus
CPH: Caffeoyl putrescine-green leaf volatile compound
DMAPP: Dimethylallyl diphosphate
GLRaV-3: Grapevine leaf roll associated virus 3
GLS: Glucosinolates
GRBaV: Grapevine red blotch-associated virus
HGT: Horizontal gene transfer
IPP: Isopentenyl diphosphate
MEP: L 2-methyl-D-erythritol 4-phosphate
MeSA: Methyl-salicylate
MVA: Mevalonic acid
PAL: Phenylalanine ammonia lyase
PMV-P: Poppy mosaic virus
PSM: Plant specialized metabolites
PVX: Potato virus X
SA: Salicylic acid
SABP2: Salicylic acid-binding protein-2
SAR: Systemic acquired resistance
SCMV: Sugarcane mosaic virus
SRBSDV: Southern rice black-streaked dwarf virus
TCV: Turnip crinkle virus
TMV: Tobacco mosaic virus
VOCs: Volatile organic compounds
